# A Case of *Escherichia coli* Hemolytic Uremic Syndrome in a 10-Year-Old Male With Severe Neurologic Involvement Successfully Treated With Eculizumab

**DOI:** 10.1177/2324709617741144

**Published:** 2017-11-06

**Authors:** Malia Rasa, James Musgrave, Keith Abe, Len Tanaka, Konstantine Xoinis, Bruce Shiramizu, Gretchen Foskett, Rhiana Lau

**Affiliations:** 1 Kapiolani Medical Center for Women and Children, Honolulu, HI, USA

**Keywords:** hemolytic uremic syndrome, Shiga toxin, Soliris, eculizumab

## Abstract

Hemolytic uremic syndrome (HUS) can be classified as typical and atypical, and the treatment recommendations currently differ between the 2 types. Eculizumab is recommended as first-line treatment for atypical HUS; however, its use in typical HUS has been controversial. We report a case of a 10-year-old male with severe neurologic impairment who was successfully treated with eculizumab, which was started 4 days after onset of neurologic symptoms. Our case supports the use of eculizumab in typical HUS with neurologic involvement, even when given later in the course, as the pathophysiology of typical HUS has been shown to involve activation of the complement pathway, similar to atypical HUS. Further studies are required to establish the efficacy and duration of eculizumab use in this patient population.

## Introduction

Hemolytic uremic syndrome (HUS) is characterized by thrombocytopenia, microangiopathic hemolytic anemia and renal failure. The typical form of HUS is most commonly caused by Shiga toxin–positive *Escherichia coli* O157:H7, contracted by ingestion of contaminated food or water and preceded by a period of bloody diarrhea. Symptoms of gastroenteritis generally begin approximately 3 days after ingestion of a contaminated source, and can include nausea, abdominal pain, and diarrhea, with bloody diarrhea in 70% of cases.^1^ The pathophysiology of HUS is thought to be due to release of Shiga toxin, which causes endothelial damage and activates the complement cascade leading to thrombotic microangiopathy that principally affects the kidney but may also involve the central nervous system (CNS), heart, liver, pancreas, and hematopoietic cells.^2-4^ Neurologic symptoms of HUS can involve seizures, coma, irritability, or confusion, and rarely may include hemiparesis or involve the cranial nerves. CNS involvement has been reported in up to 20% of cases of typical HUS, and presentation with severe CNS manifestations is associated with increased mortality and long-term neurologic damage.^5^ HUS is a complication of *Escherichia coli* and *Shigella* infection in less than 10% of cases, which may suggest that there remain other unknown factors that are likely involved in the development of HUS.^4^

Hemolytic uremic syndrome may also be caused by streptococcus pneumoniae, most often following invasive pulmonary infection. The pathogenesis is thought to involve exposure of the Thomsen-Friedenrich antigen by a neuraminidase producing pathogen, such as pneumococcus.^1^

Atypical HUS (aHUS) is not usually preceded by a bloody diarrheal illness as in typical HUS, and is most often caused by inherited or acquired dysregulation of the alternative complement pathway leading to abnormal activation.^6^ However, an infectious event such as gastroenteritis with diarrhea or pregnancy can trigger aHUS secondary to dysfunction of the complement system.

Many recent studies have confirmed the efficacy of eculizumab in the treatment of aHUS and demonstrated that treatment with eculizumab early in the course of illness improves preservation of renal function.^7-9^ Eculizumab is an anti-C5 monoclonal antibody that acts by preventing cleavage of C5, thereby preventing the formation of the membrane attack complex, which is composed of C5b-C9.^10^ It is currently approved for adult and pediatric patients for treatment of aHUS; however, its use in typical HUS is not well established. Treatment for typical HUS currently involves symptomatic therapies that are directed at preventing complications secondary to renal failure. A study performed in Germany during a large outbreak of EHEC O104:H4 did not find a positive effect of eculizumab for patients^11^; however, other case reports have recently shown eculizumab to be successful in the treatment of typical HUS.^12,13^

In this article, we describe a 10-year-old male with typical HUS with renal and severe neurologic involvement successfully treated with eculizumab. This case lends support to the use of eculizumab in severe cases of typical HUS, even when given later in the course of the disease, which has previously been reported to be less beneficial.^11,13,14^ Informed consent for patient information to be published in this article was not obtained because the Hawaii Pacific Health Research Institute determined that approval was not required (Study Number: 2017-046).

## Case Report

A 10-year-old male was transferred to our facility from an outside hospital with a 4-day history of bloody diarrhea, thrombocytopenia, anemia, and elevated blood urea nitrogen (BUN) and creatinine with concern for HUS. On admission, significant laboratory findings supporting a diagnosis of HUS included the following: hemoglobin 10.5 g/dL, platelets 45 × 10^9^/L, BUN 43 mg/dL, creatinine 2.46 mg/dL, and lactate dehydrogenase of 5455 U/L. Stool culture grew out *E coli* O157:H7, classifying his case as typical HUS, although Shiga 1 and 2 toxins were not detected. Complement factors (C3, C4, CH50, complement factors H, B, and I) and genetic panel for aHUS genetic mutations were obtained and were pending during his hospitalization and eventually returned as normal.

Over the course of the first 2 days of his hospitalization, BUN and creatinine increased and a hemodialysis catheter was placed in anticipation of starting hemodialysis, which was initiated on day 3.

In the evening after hemodialysis, he complained of numbness and tingling of the left arm and cheek and had dysarthric speech. His neurologic symptoms waxed and waned over the next 2 days. On day 7, he had worsening left facial droop with left sided weakness and required noninvasive CPAP (continuous positive airway pressure) by mask for respiratory support.

A neurology consult was obtained and on exam found confusion with inability to follow directions, left sided hemineglect, limited visual fields on the left, and a moderate left hemiparesis. Further evaluation with brain MRI (magnetic resonance imaging) and magnetic resonance angiography showed no abnormalities. Electroencephalogram was abnormal with encephalopathic background activity due to poor organization and excessive slowing of background activity, but had no electrographic seizure activity or epileptiform discharges.

His BUN and creatinine continued to rise over this time (peak of 102 mg/dL and 7.36 mg/dL, respectively), and with worsening of his clinical picture, he was placed on continuous renal replacement therapy (Prismaflex System, CVVHDF mode with HF1000 hemofilter, Baxter Healthcare Corporation, Deerfield, IL) from day 6 through day 12 of the hospitalization.

**Figure 1.**
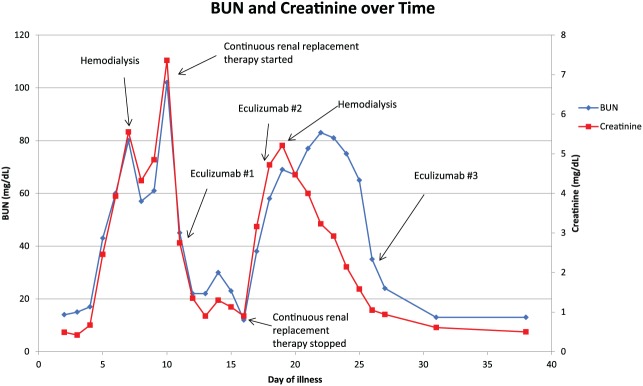
The trend of BUN and creatinine over the course of the patient’s illness.

**Figure 2.**
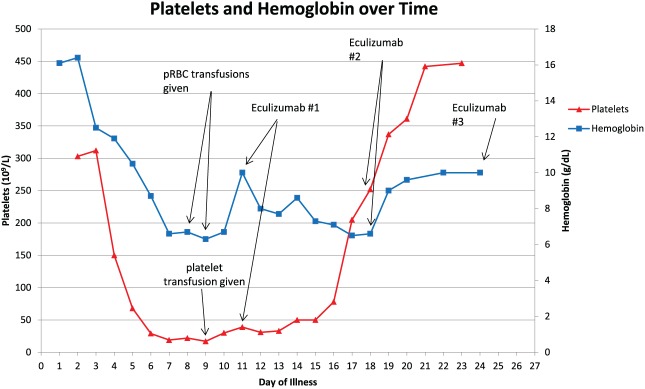
Platelets and hemoglobin over the course of the patient’s illness.

After discussion between the pediatric intensivists, neurology, and nephrology consultants involved in his care, the decision was made to start eculizumab 600 mg (based on weight), 4 days after first manifestation of neurological symptoms, which was day of illness 11 and hospital day 7. He was vaccinated with both meningococcal B and Menactra vaccinations prior to the start of eculizumab therapy, and was started on penicillin prophylaxis. On day 10 of the hospitalization, he had 2 brief generalized tonic-clonic seizures and was given lorazepam and started on leviteracetam for antiseizure therapy. He had no further seizures throughout his hospitalization and his neurologic deficits started to improve the following day (4 days after the initial dose of eculizumab). Urine output started to improve at about day 14 of the hospitalization, or 1 week after his first dose of eculizumab. He was given a total of 3 eculizumab doses at 1-week intervals, the first 2 doses at 600 mg and the final dose at 900 mg. In total, he required 2 hemodialysis sessions, one session prior to starting continuous renal replacement therapy (CRRT) and another after discontinuing CRRT. His BUN and creatinine gradually improved and were 24 mg/dL and 0.94 mg/dL, respectively, by discharge. Urine protein to creatinine ratio showed downward trend throughout his hospitalization and had dropped from 30, 160 mg TP/g Cre at its peak to 1091 mg TP/g Cre prior to discharge. At the time of discharge minimal residual neurological deficits were noted, mainly with a mild hemiparesis and some mild cognitive challenges. Both BUN and creatinine were within normal limits on recheck 4 days after discharge at 13 mg/dL and 0.61 mg/dL, respectively. The trend of BUN with creatinine and hemoglobin with platelets are shown in Figure 1 and Figure 2, respectively.

On outpatient follow-up with nephrology, he was started on low-dose lisinopril for mild proteinuria, with subsequent urinalysis showing microscopic hematuria and otherwise normal urine specimen. In neurologic and rehabilitation follow-up 2, 8, and 14 months after onset of his illness, his neurologic symptoms had resolved completely and he was able to perform all activities of daily living independently.

## Discussion

In our case of typical HUS with renal and CNS involvement, the use of eculizumab was associated with a dramatically positive neurologic outcome with complete resolution of neurologic symptoms and improvement in kidney function. This case report echoes positive findings previously published that support further investigation into the use of eculizumab in typical HUS.^12-15^ Eculizumab was not found to affect the clinical course of a German cohort during an outbreak in 2011;^11^ however, in this study, eculizumab was administered days to weeks after onset of disease and neurologic complications, and it has been suggested that complement activation in the setting of Shiga-toxin enterocolitis infection may resolve within a week.^16^

Typical HUS has been shown to activate the complement pathway directly,^4^ which may explain why eculizumab has been successful in treating typical HUS as eculizumab acts to block the formation of the membrane attack complex, thereby inhibiting the complement cascade. Shiga toxin plays a key role in the pathophysiology of typical HUS, though testing results were negative in our case. Previous studies have discussed the possibility that Shiga toxin–negative *E coli* cases of HUS may develop from Shiga toxin–positive *E coli* that lose their stx genes during the course of infection^17^; thus stool studies performed even a few days after onset of symptoms may result negative secondary to the loss of stx genes. Stool culture in our patient was taken within 1 day of onset of symptoms of diarrhea, vomiting, and abdominal pain; however, as previously described, symptoms of *E coli* infection may occur up to 3 days after ingestion of contaminated source, thus there is a possibility that the stx genes may have already been lost by the time of collection. The question of pathogenicity of strains of *E coli* that lose the stx gene early in infection has been discussed; however, no conclusions could be drawn in regard to the risk of developing HUS from such a strain.^18^

Gitiaux et al reported 7 patients with HUS who had significant MRI findings, particularly diffusion abnormalities in the basal ganglia and white matter, which resolved on 6-month follow-up.^15^ Pape et al found that treatment with eculizumab was most beneficial when initiated within hours of the development of neurologic symptoms.^13^ Our patient was treated 4 days after the onset of waxing and waning neurologic symptoms and 1 day after persistent neurological symptoms, but prior to the development of seizures, and he subsequently had complete recovery of neurologic function. Our patient had good neurologic outcome with eculizumab as well, although his brain MRI did not have the reduction of apparent diffusion coefficient (abnormal restricted diffusion) as seen in the patients in their study. This suggests that eculizumab given later in the course of disease may still be effective. However, given that this was an isolated case, we are unable to extrapolate this patient’s rapid marked improvement of neurological, renal, and hematological abnormalities into a broader hypothesis that eculizumab can be beneficial even when given days after the first onset of neurologic symptoms.

As previously discussed, the development of neurologic symptoms with HUS is associated with increased disease severity, affecting up to 20% of patients with HUS.^5^ This patient had a fairly severe initial course including renal failure, hemiparesis, encephalopathy, visual field deficits, and seizures. Eculizumab is proposed to improve neurologic outcome by preventing complement-mediated pathology, particularly thrombotic microangiopathy. Side effects of eculizumab include infection, given that it inhibits the formation of the complement membrane attack complex. Our patient did not suffer any severe bacterial infections within 6 months after treatment with eculizumab and received prophylactic vaccination for meningococcus and penicillin prophylaxis prior to eculizumab administration. Several studies have been performed to assess the safety of eculizumab in aHUS patients, and it has been found to be well tolerated in patients with aHUS with no meningococcal infections or cumulative toxicity noted.^8,19,20^

In conclusion, the use of eculizumab for treatment of typical HUS with neurologic involvement may be beneficial in preventing long-term neurologic sequelae. In our case, we saw improvement in the patient’s neurologic condition despite initiating eculizumab 4 days after the onset of neurologic symptoms. Previous reports had found that late treatment with eculizumab was not as beneficial^11,13,14^; however, our patient had complete resolution of neurologic involvement. The therapy for typical HUS currently focuses on symptomatic care and there is no definitive treatment for neurologic symptoms. Eculizumab is currently recommended as first-line treatment for aHUS, and it has shown to have a positive effect on long-term renal function. Further studies on a larger number of patients could help establish efficacy and determine the optimal dosage and number of doses that may be given for adequate treatment of typical HUS with eculizumab.
